# Supplementary material for: Are carbon emissions trading and green financial instruments synergistic? -Comprehensive quantitative research based on content analysis
Source: PLoS One. 2024 Mar 7;19(3):e0298601. doi: 10.1371/journal.pone.0298601 (PMC10919601; doi:10.1371/journal.pone.0298601)
Supplement: S1 Data — (ZIP) [file pone.0298601.s001.zip › Data/地级市Z_F工作报告.zip!/╜¡╦╒/╤╬│╟╩╨/└┤╫╘╣1⁄2╓┌║┼í╛┬φ┐╦╩2╛▌═°í┐_2013.docx]

各位代表:

现在,我代表盐城市人民政府向大会作工作报告,请予审议,并请各位政协委员和其他列席人员提出意见。

2012年工作回顾

过去的一年,是新一届政府的开局之年,也是我市继续抢抓机遇、克难求进,推动经济社会又好又快发展取得较好成绩的一年。预计全市完成地区生产总值3120亿元,增长12.7%;公共财政预算收入312.8亿元,增长16.3%;城镇居民人均可支配收入22520元,增长16%;农民人均纯收入12000元,增长14.2%。主要经济指标增幅位居全省前列,以市为单位总体上达到省定全面小康标准,县域全面小康进程苏北领先,较好地完成了市六届人大五次会议确定的目标任务,落实了市七届人大一次会议提出的工作要求。

一、积极应对各种困难和挑战,实现了经济平稳较快增长。受国内外宏观经济环境变化影响,我市经济发展遇到前所未有的困难,经济下行压力自始至终存在。我们坚持定期分析研究经济形势,主动采取各项扎实举措组织推进经济工作。力保企业健康运行。深化细化“三服务”,强化政策支持和要素保障,制定落实企业减负措施,与企业共度难关、共克时艰。全市规模以上工业实现增加值1354亿元,增长17.3%。东风悦达起亚公司产销汽车48万辆,累计突破200万辆。狠抓项目推进。实施八大类重点工程、工业“三百”工程,组织重点项目“家家到”观摩,下半年开始每月10日举行重大项目集中开工活动,东风悦达起亚汽车第三工厂等一批项目顺利推进。全年完成固定资产投资1938亿元,增长22.1%,其中工业投资1237亿元,增长20.6%。发展民营经济。认真落实国务院、省政府鼓励引导民间投资各项政策,推动全民创业。全市新发展私营企业3.1万家,个体工商户18万户,民营经济主要指标增速继续保持全省领先。积极争取支持。抢抓国家稳增长政策预调微调机遇,围绕项目审批、政策支持,主动到国家和省对接争取。积极申报国家可持续发展实验区,国家珍禽自然保护区区划调整方案获得批准,省政府出台支持盐城发展专项政策,为我市推进科学发展注入新的活力。

二、坚定不移调整产业结构,推动了经济转型升级。全市三次产业结构调整为14.4:47.5:38.1,二三产业比重提高0.6个百分点。发展壮大新特产业。加大战略性新兴产业规划提升、项目推进力度,强化特色产业园区建设,发展高新技术产业,动员市直部门支持服务新特产业发展,强力推进“千百十”工程、大企业(集团)培育工程。成功举办首届国际汽车博览会、环保产业博览会。全市新特产业实现开票销售1700亿元,增长26%。实施服务业提速计划。积极推进服务业“双百”工程,抓好30个重点服务业集聚区建设,先锋国际广场一期建成运营。阜宁金沙湖景区获批省级旅游度假区。全市实现服务业增加值1190亿元,增长12.9%;社会消费品零售总额1024亿元,增长14.4%。加快发展现代高效农业。部署实施农业现代化工程,继续加强“三大载体”建设,提升农业产业化水平。全市新增高效农业面积76万亩,高效农业总面积占比达到54%。粮食生产实现“九连增”,被表彰为全国粮食生产先进市。新认定省级现代农业(渔业)示范园区8个、省级定点批发市场7个。加强节能减排和环境保护。坚持源头控制、科技支撑、严格监管,较好地完成了节能减排任务。加快绿色盐城建设,全市新增造林折实面积31.8万亩,森林覆盖率达到20%,建湖县建成全国绿化模范县。开展美好城乡建设行动,完成1374个村庄环境整治任务。

三、全力主攻沿海重大项目,加快了沿海发展步伐。强势推进港口建设。支持大丰港区发展,建成万吨级泊位4个,刘大线疏港航道建成通航。滨海港区中电投煤炭码头开工建设,响水灌河2万吨级公用码头建成使用,射阳港区南北导堤顺利竣工,东台深水港建设预可行性报告通过专家组论证。盐城港货物吞吐量突破3000万吨。积极实施临港产业项目。列入省沿海开发五年推进计划的78个项目,已实施65个,累计完成投资1299亿元。全市沿海发展50个重点项目,完成投资450亿元。陈家港电厂一期工程获国家发改委核准,江苏博汇项目完成投资160亿元,大丰港经济区获批省级开发区。开发利用滩涂、盐田资源。东台条子泥匡围一期工程条南围区正式合龙。对盐田资源开发利用进行专题调研部署,组建灌东、新滩开发公司。口岸、海关、检验检疫、海事、边防在服务沿海发展中发挥了积极作用。

四、扎实抓好城市和基础设施建设,改善了城乡发展条件。全力推进中心城市建设。组织实施市区十大类147个城建项目,完成投资133亿元。着力提升城市功能,串场河景观带两侧道路基本贯通,金融城一期工程开工建设;亭湖高级中学一期、市第三小学等5所学校建成招生;市新图书馆、广播电视塔投入运行;盐城大桥实现全幅通车。支持新区发展,城南新区积极打造智慧新城、生态新城、现代新城,市开发区新城拉开框架,亭湖新城、盐都新城加快建设。加大老城区改造力度,强化城市综合管理。大力实施“露水增绿”工程,国家园林城市创建通过省级验收。加快县(市)城和重点镇建设步伐。对镇域发展实行分类指导考核。全市城市化率达到55.5%,提高1.5个百分点。扎实推进城乡统筹,完善提升36个试点镇村建设规划,139个重点项目完成投资60多亿元,涌现出一批各具特色的新镇区、新社区、新村庄,东台梁垛镇临塔村获得“江苏最美乡村”称号。加大交通基础设施建设力度。开工建设阜建高速公路,加快实施临海高等级公路。全市新开通一级公路120公里、二级公路150公里、农村公路323公里,改造农村公路桥梁278座。盐城南洋机场改扩建工程顺利推进,新增两条国际国内航线。强化农田水利建设和农业综合开发,疏浚整治县乡河道512条,新建高标准农田65万亩。实施万顷良田建设工程,推进土地整治。加强沿海电网规划建设,邮政、通信设施建设等取得新的进展。

五、进一步深化改革扩大开放,激发了经济社会发展活力。深入推进各项改革。实施营业税改征增值税试点工作。深化医药卫生体制改革,抓好公立医院改革试点,巩固完善基本药物制度。加快文化体制改革,组建市演艺集团,筹建市广电集团。深化农村各项改革,新发展农民专业合作社848家,新增土地流转面积43.4万亩。着力提升开放水平。精心组织一系列重大招商活动,成功举办“5•18”、“11•18”经贸洽谈会,实施了一批利用外资重点项目,全市新批投资3000万美元以上外资项目39个。注册外资实际到帐21.4亿美元,增长26.9%。外贸出口总额35.8亿美元,增长20%。推动开发区转型升级,盐城综合保税区实现封关运行,全市开发区业务总收入增长42%。不断强化科技创新。支持企业建立研发平台,加快城南科教城规划建设,深化政产学研金合作,全社会研发投入占地区生产总值比重达1.4%。开展“国家知识产权试点城市”建设,全年专利申请量突破1.4万件。新增中国驰名商标6件。全力打造长三角北翼人才高地、金融高地,在上海成功举办第二届沿海发展人才峰会,在南京成功举办沿海项目金融对接会,金融高地建设规划通过评审,新引进银行保险证券机构10家,新增股权投资等公司12家,全市新增贷款289.6亿元。

六、千方百计保障改善民生,提高了人民群众生活水平。多渠道扩大就业。全面落实促进就业、鼓励创业各项政策,全市新增城镇就业10.5万人,帮扶4600名就业困难人员再就业,城镇登记失业率控制在2.5%以内。完善社会保障体系。各类保险持续扩面,城乡居民养老保险并轨运行。落实低保、五保、优抚救助自然增长机制,及时发放尊老金、养老服务补贴以及困难家庭物价上涨动态补贴。开展新一轮扶贫开发行动,帮助16万低收入人口脱贫。积极兴办民生实事。20件实事项目基本完成年度任务。建成盐龙湖饮用水源工程,大市区区域供水管网基本实现乡镇全通达。扩大住房保障范围,市区新建各类政策性保障住房100万平方米。改善老城区人居环境,完成66条后街巷道、6个老旧小区整治任务。推进校安工程,全市新建改造校舍115.5万平方米。积极筹集资金1.6亿元,基本还清16年前的新长铁路借资款。落实临时救助和特殊困难残疾人救助政策,累计发放救助资金8000万元,免费为困难群众提供法律援助5780件。

七、大力强化社会建设与管理,促进了社会和谐稳定。全面发展各项社会事业。推进教育事业优质均衡发展,全市新建中小学、幼儿园70所。建立健全基层医疗卫生服务体系,市区新建3个社区卫生服务中心、4个社区卫生服务站,亭湖区人民医院投入运营。实施文化惠民工程,240个农家书屋实现数字化阅读,13个文化产业集聚区建设初见成效。深化群众性精神文明创建活动,有7人和1个团队入选“中国好人榜”。全民健身运动蓬勃开展,青少年校园足球进入全国先进行列,骆晓娟荣获奥运会女子重剑团体金牌,实现我市奥运冠军零的突破。加强人口与计划生育工作,人口自然增长率控制在3.5‰以内。注重发挥工会、共青团、妇联、科协等群众团体作用,关工委、老龄、红十字、慈善等事业进一步发展,物价、供销、气象、防震减灾、住房公积金、新闻出版、档案、地方志、人事编制、机关事务管理、保密、援疆等工作取得新的进展。实施社会管理创新工程。创新社区管理办法,推行网格化管理,完善了社会管理工作体系。加强“平安盐城”、“法治盐城”建设,深入开展“四项排查”、“三解三促”活动,排查化解各类社会矛盾纠纷。全市化解信访积案590件,实施重大项目风险评估130件,整改安全环保治安隐患1.2万个。被省委、省政府表彰为社会管理综合治理先进市。强化食品药品监管、消防、民族和宗教事务等工作,推进国防动员、国防后备力量建设,优抚安置、拥军优属、军民共建取得新的成绩,海军一艘护卫舰被命名为“盐城舰”,荣获“全国双拥模范城”四连冠。

八、切实加强政府自身建设,增强了政府执行力和公信力。深入开展“迎接十八大、保持纯洁性”教育实践活动,完善政府工作规则,制定加强政府自身建设意见。强化政府服务职能。深化行政审批制度及公共资源交易体制改革,成立市政务服务管理办公室,组建市公共资源交易中心,建成“12345”公共服务平台。实施新一轮行政审批权限下放,对市级机关53个职能部门许可审批和收费事项进行了集中清理。提高依法行政水平。加强法治政府建设,自觉接受市人大及其常委会、政协以及各民主党派、社会团体、人民群众的监督,认真听取人大代表、政协委员反映社情民意,办理市人大代表建议314件、市政协委员提案446件。全面推进行政权力网上公开透明运行,建立网络新闻发言人制度,不断提高统计、审计、行政监察工作水平。加强廉政建设。严格落实党风廉政建设责任制,完善惩防体系,健全招投标、政府采购等重点领域的反腐倡廉长效机制。大力倡导艰苦奋斗,坚决制止铺张浪费。加强廉政文化建设,强化公务员教育管理,严肃查处违法犯罪行为,树立了政府为民务实清廉的良好形象。

各位代表,过去的一年,在严峻复杂的宏观环境下,全市经济社会发展能够取得上述成绩,实属不易。这是省委、省政府和市委正确领导的结果,是各地、各部门、各单位同舟共济、拼搏奋进的结果,凝聚着全市人民的智慧和力量。在此,我谨代表市人民政府,向全市人民,向人大代表、政协委员,向各民主党派、工商联、人民团体和各界人士,向驻盐人民解放军和武警官兵、省属驻盐各单位,向关心和支持盐城发展的各位老同志、海内外朋友,表示崇高的敬意和衷心的感谢!

各位代表,在肯定成绩的同时我们也清醒地看到,我市经济社会发展中还存在许多困难和问题:产业结构仍不够合理,新兴产业尚未形成规模优势,中小企业发展面临生产经营成本上升、效益下降等突出问题;沿海重大产业项目不多,港城建设推进不快;城市基础设施、功能配套建设欠账较多,城镇化水平亟待提升;农业现代化基础薄弱,村级负债较重;改善民生、维护社会稳定的任务还很艰巨;机关作风不实、办事拖拉、推诿扯皮等现象依然存在,政府服务效能需要进一步提高。对这些问题,我们一定高度重视,并在今后的工作中采取更加有力有效措施,切实加以解决。

2013年工作安排

今年我们面临的形势依然严峻。全球经济虽然存在温和回升迹象,但宏观环境仍然错综复杂、充满变数;区域竞争更加激烈,进位争先难度进一步加大;困难矛盾有增无减,改革发展稳定的任务更为繁重。在清醒认识不利因素的同时,我们更要看到许多积极变化和有利因素。今年是全面贯彻落实党的十八大精神的第一年,用十八大精神武装头脑、统一思想、指导实践,有利于我们凝聚各方面智慧力量,加快建设高水平小康社会;今年是各种机遇政策效应全面释放之年,沿海开发、长三角一体化发展、省振兴苏北“一市一策”支持等多重机遇叠加,有利于我们乘势而上,实现科学发展新跨越;今年是盐城建市30周年,经过近30年的努力,我市工业化、城镇化已经进入加速期,经济综合实力和抗风险能力大为增强,有利于我们在新的起点上,向着更高目标迈进。为此,我们一定要科学研判形势,切实增强忧患意识、机遇意识、责任意识,坚持“苏北领先、全省争先”发展定位,坚定信心、攻坚克难,抢抓机遇、赢得优势,扎实推进新型工业城市、统筹发展城市、创新创业城市、生态文明城市、和谐幸福城市建设,奋力开创盐城科学发展新局面。

今年政府工作的总体要求是:全面贯彻落实党的十八大精神,以邓小平理论、“三个代表”重要思想、科学发展观为指导,以“推进科学发展、建设美好盐城”为主题,以转变发展方式为主线,以提高经济增长质量和效益为中心,以全面建成高水平小康社会、实现盐城科学发展新跨越为目标,深入实施“八项工程”,全力推进经济较快增长、沿海加快开发、产业转型升级、城乡统筹发展、改革开放创新、生态文明建设、民生持续改善,稳中求进,真抓实干,努力实现经济又好又快发展和社会和谐稳定。

全市经济社会发展主要预期目标是:地区生产总值增长12%,公共财政预算收入增长16%,固定资产投资增长22%,社会消费品零售总额增长15%,外贸出口总额增长15%,全社会研发投入占地区生产总值比重1.9%左右,城镇居民人均可支配收入、农民人均纯收入增长13%,居民消费价格涨幅控制在3.5%左右,城镇登记失业率控制在4%以内,节能减排完成省定目标,人口自然增长率控制在4‰左右。

围绕上述总体要求和目标任务,我们将着力抓好以下六个方面工作:

一、强化又好又快导向,保持经济持续健康较快增长。稳增长是今年经济工作的突出任务。要努力实现实实在在、没有水分的增长,尊重经济规律、有效益、有质量、可持续的增长,群众认可、得实惠的增长。

大力推进新型工业化。坚持以工兴市第一方略不动摇,在加快发展中推动转型升级,在做优存量的同时做大增量,扶持实体经济发展壮大。深入推进重大项目建设,继续优化投资结构,扩大有效投入,充分发挥投资对经济增长的关键作用。集中精力、扎实推进工业“三百”工程,强化项目服务、要素协调、督查考核,促进重点项目加快建设、达产达效,成为新的经济增长点。深入推进“千百十”工程,落实扶持政策、服务措施,推动现有企业技改扩能、合作重组、上市融资,实现转型发展、创新发展,提升企业核心竞争力。继续实施工业大企业(集团)培育工程,支持中小企业发展,帮助解决融资、用工、技术、市场等难题,促进企业转型升级、做大做强。深入推进新特产业发展,深化“四有三聚”思路和办法,重抓新能源、节能环保、新能源汽车、海洋生物等战略性新兴产业和一批特色产业,完善“一业一策”政策措施,努力抢占产业发展制高点。深入推进传统产业改造升级,积极运用新技术、新工艺、新装备、新材料,改造提升机械装备、纺织、化工等传统产业,加快发展高新技术产业,推动信息化与工业化深度融合,提升产业整体竞争力。

全力加快沿海开发。坚持规划引领、“三港”联动、“五重”并进,整合资源,加快发展,使沿海地区成为活力最强、增长最快的经济板块。港口码头建设上,大力推进大丰港区三期通用码头、滨海港区煤炭码头、射阳港区通用码头建设和响水灌河航道整治工程,盐城港完成建设投资56亿元,货物吞吐量突破5000万吨。沿海港城开发上,进一步加快核心区建设,完善公益性、功能性设施配套,实施供水、供热、供气、通信、消防、污水处理等配套工程,完成投资130亿元,展示港城新貌。临港产业发展上,大力引进一批龙头型、基地型、临港型重大产业项目,加快汽车制造、新能源、新装备、新材料等特色产业基地建设。开工建设三鼎石化、中海油LNG、海上风电等项目,全力推进东风悦达起亚汽车第三工厂、中汽中心盐城汽车试验场、江苏博汇、华锐风电装运基地等项目,积极做好德力西精铜科技产业园、联孚石化重油裂解等项目推进工作,增创沿海产业发展新优势。载体平台打造上,全面启动国家可持续发展实验区建设,推动沿海经济区创建省级开发区,开展国家级百万亩滩涂综合开发试验区规划编制、申报工作,加快国家海上风电装备技术研发中心、科技兴海示范基地建设。实施灌东、新滩两大盐场综合开发,拉开启动区建设框架。体制机制创新上,重点落实省政府支持盐城沿海发展“两区两产五港城”专项政策,引进境内外战略投资者参与沿海开发,加快推进市开发区大丰临港产业园建设,推动市本级和内陆县份深度融入沿海开发,形成沿海发展合力。

积极发展现代服务业。继续实施服务业提速计划,促进现代服务业与先进制造业融合发展。加快发展现代物流、金融、商务、汽车和节能环保服务、软件及服务外包等生产性服务业,支持企业由产品制造商向技术研发商、工程服务商转变。更大力度推动金融业创新发展,扩大信贷规模,推进直接融资,建立“盐创母基金”,强化国有投融资平台建设管理,加快金融城建设。优化金融生态环境,加强监管,防范风险,着力打造金融高地。提升发展商贸、餐饮、旅游、家庭服务等生活性服务业,开拓连锁经营、电子商务等新型业态,推进沿海湿地旅游经济区、丹顶鹤湿地生态公园、麋鹿栖息地湿地景观修复工程建设,努力扩大消费需求,增强消费对经济增长的拉动作用。继续推进30个服务业集聚区建设,突破100个重点项目,培植100家龙头企业,打造服务业特色品牌,促进服务业集聚区量质并举、加快发展。

二、坚持城乡统筹建设,推动城乡互动发展共同繁荣。城镇化是加快推动城乡发展一体化的重要抓手。要坚持从实际出发,科学规划,因势利导,积极推进城乡统筹“六个一体化”,推动城镇化与工业化、农业现代化协调发展。

进一步做大做强中心城市。按照完善功能、拓展新区、改善民生、打造特色的总体要求,全力推进“八大组团”建设。建军路组团重点抓好道路环境整治、商业大厦改造以及地下商业街、同曦鸣城等项目建设,着力打造大市区核心商业圈。聚龙湖组团重点抓好金鹰天地、凤凰文化广场、紫薇国际广场等城市综合体建设,金融城一期完成主体工程。龙湖组团重点抓好龙湖湖体开挖,启动周边基础设施、配套设施建设。亭湖新城组团重点抓好环保、光电产业园基础配套以及城市功能性项目建设。亭湖新洋新城组团重点抓好基础设施、公共配套设施建设。盐都新城组团重点抓好神州路商业街区、物流园区核心区、东方汽车广场及拓展区、市民中心建设。市开发区新城组团重点抓好汽车服务集聚区、商贸中心、步凤内河港等项目建设。火车站组团重点抓好城市交通枢纽中心、区域商业中心建设。继续实施“露水增绿”工程。大力推进串场河景观带、通榆河生态走廊、城南小新河景观带以及龙湖公园、天山公园、青年公园、民俗园等“十大公园”建设,扩大绿量,提升品质,创成国家园林城市。推进蟒蛇河、小洋河、越河等市区河道整治,进一步彰显水绿盐城特色。着力增强城市综合功能。实施范公路快速路、世纪大道东延、东进路西延、青年路跨宁靖盐高架大桥等道路桥梁工程,合理布局建设市区学校、文化休闲、农贸市场等公共场所。围绕建设“智慧盐城”,组织实施政府信息资源中心等重点项目。全面提升城市建设管理水平。注重调动区、镇(街道)、社区积极性,建立重心下移、责权利相配套的城市建设管理新体制。加强网格化、精细化、长效化城市管理体系建设,深入开展市容环境、交通秩序整治,提高城市文明程度。设立城乡社区建设引导资金,大力支持社区服务平台、社区工作者队伍建设,增强社区服务功能。

扎实推进农业现代化工程。围绕建设现代农业强市目标,组织实施四大类100个重点项目,大力发展生态、高效、外向、特色农业。着力抓园区、扩规模。推进东台、建湖国家级现代农业示范区建设,加快伍佑生态高效农业示范园等10个现代农业园区发展,推广应用农业新品种、新技术、新机具,培大育强蔬菜、生猪、家禽、林业等“双百亿”优势产业。着力抓加工、强龙头。充分发挥我市农业资源优势,强化招商引资,积极引进重大产业化项目,培大育强现有龙头企业,推动农产品加工集中区建设,促进农产品加工业快速发展。着力抓市场、活流通。推进市县两级农副产品市场建设,发展网店直销、农产品交易所、物流网等新型业态,健全农产品市场网络体系。实施农产品品牌战略,完善农产品质量安全体系。着力抓改革、促发展。深化农业经营体制改革,培育农民专业合作组织,全面推进农村集体土地确权登记发证,积极稳妥推进土地流转,抓好东台国家农村金融试验区建设。认真落实强农惠农富农政策措施,加强水利建设,实施万顷良田、高标准农田建设、黄河故道综合开发工程,夯实现代农业发展基础。扎实推进村级组织“四有一责”建设,壮大农村集体经济,逐步化解村级债务。

加快新型城镇化步伐。准确把握城镇化内涵,提高城镇化质量,着力增强城镇综合承载能力。加快中小城市建设。围绕建设现代化中等城市,同步推进新区建设、老城改造,加强基础设施、公共配套设施建设,增强产业发展、公共服务、吸纳就业、人口集聚功能,把各县(市)城做大做强做美。加快生态小城镇建设。坚持科学规划引领,节约集约利用土地,深化户籍制度改革,吸引农民进镇入住、就业创业,发展镇域经济,着力打造一批具有产业特色、乡村特点、生态特质的小城镇。加快康居示范村建设。继续实施美好城乡建设行动,加大农村环境综合整治力度,着力打造一批康居示范村。扎实推进36个城乡统筹试点镇村建设。

加强城乡交通基础设施建设。围绕构建现代化综合交通运输体系,开工建设连盐铁路盐城段,做好新长铁路盐城至海安段电气化改造、徐宿淮盐铁路盐城段、盐泰锡宜城际铁路盐城段前期工作,推进阜建高速公路、331省道盐城西段、348省道阜宁段工程建设,临海高等级公路基本建成通车,盐淮高速盐城至大丰港段开工建设。通榆河东台南段建成通航。完成南洋机场扩建工程。实施农村公路等级化及危桥改造工程,完善交通安全设施,推动所有行政村开通客运班车。扎实抓好电力、天然气、通信、邮政等基础设施建设,逐步实现城乡基础设施共建共享。

三、深入推进开放创新,不断增强创新创业动力活力。改革开放是实现盐城科学发展新跨越的必由之路。要坚持以改革促发展,以开放促创新,充分激发全社会创新创业热情,形成推动发展的强大合力。

坚定不移扩大对外开放。更大力度实施经济国际化战略,推动开放型经济全面提升、重点突破。进一步提高招商引资质量。突出围绕新特产业发展,面向重点地区,加快建设高素质的专业化招商队伍,强化龙头型、产业链项目招商,积极打造江苏韩资密集区、沿海台资新高地。优化投资环境,扩大利用外资领域,提高利用外资质量。举办好汽车、环保、新能源产业博览会。进一步加大外贸转型升级力度。鼓励更多企业开拓国际市场,千方百计扩大外贸总量。优化外贸出口结构,加强自主出口品牌、出口基地建设,提高出口产品竞争力。推进盐城综合保税区项目建设。进一步加快“走出去”步伐。支持拥有一定规模、品牌和核心竞争力的龙头企业开展跨国投资与经营,在参与国际竞争中发展壮大。

坚持不懈实施创新驱动。把科技创新摆在优先发展的战略位置来抓,加快建设创新型城市。提升企业创新创业能力。更加突出企业创新主体地位,引导创新政策、创新资源、创新人才向企业集聚,支持规模以上工业企业、高新技术企业全面建立研发机构,实施企业知识产权战略,打造创新企业集群。组织开展中小企业科技创新创业行动,评选科技创业企业家。强化创新创业平台建设。推进开发区、新特产业园区加强公共服务平台、科技研发平台建设,促进各类园区向创新型园区转型。推进城南科教城、市开发区科技绿洲等重点项目建设。推进政产学研金更加紧密结合,支持东台建设省级科技金融合作创新示范区。引进培养创新创业人才。围绕打造人才高地,引进更多高层次人才和创新创业团队,培养更多高技能实用型人才。实施“双创”领军人才集聚工程、科技型企业家培育工程,举办第三届沿海发展人才峰会,优化人才发展环境,充分发挥各类人才作用。

坚决主动推进园区开发。强化开放创新意识,促进开发园区转型升级、跨越发展。推进开发区“二次创业”。围绕开发区产业定位,突破重大项目,努力把开发区建成科学发展先导区、产业集聚示范区。加强开发区生产、生活、生态等各类功能建设,促进产城融合。大力推动现代汽车名城建设,发展汽车及零部件配套产业,打造中国乃至世界重要的汽车零部件生产基地。盐城高新区争创国家级高新区,环保产业园争创省级高新区。推进特色产业园区集聚发展。坚持专业化、特色化方向,进一步优化开发区产业布局,采取“一区多园”发展模式,加快建设区中园。鼓励产业链配套企业向特色园区集聚,形成企业集群。整合提升乡镇工业园区,加强特色园区品牌创建工作。推进园区合作共建。进一步加强与上海、苏州、无锡、常州等地区的园区合作,主动承接长三角地区产业转移,培植新的经济增长点。

四、加强生态文明建设,全面促进资源节约环境友好。加强生态文明建设比以往任何时候都更为紧迫。今年要在推动经济绿色转型、改善人居环境等方面取得新的突破。

推动绿色发展。牢固树立尊重自然、顺应自然、保护自然的理念,深入实施生态文明建设工程。扶持发展新能源、节能环保产业,加快发展生态旅游、生态农业,构建以低碳为特征的现代产业体系。推进生产、流通、消费各环节循环经济发展,大力推广资源节约、替代和循环利用技术,实现资源高效利用、经济永续发展。省级以上开发区全面开展生态工业园建设。扎实抓好节能减排,深入开展第三轮化工生产企业专项整治行动、万家企业节能低碳行动,严把新上项目节能评估关、环保准入关,淘汰落后产能,削减存量、控制增量,确保完成省下达的节能减排目标。

改善生态环境。启动实施新一轮清水走廊建设行动,扎实推进饮用水源安全保障工程,加快市区饮用水源预警水质自动站建设,完成水源保护区内企业关闭、居民搬迁工作。落实以河长制为核心的水源保护责任制,全力保障水环境安全。深入实施蓝天工程,严格控制工业废气污染,开展油气回收、挥发性有机污染物整治,建立城市扬尘污染防治机制,规范机动车环保标志发放和管理,加强农作物秸秆综合利用,全面改善大气环境质量。切实抓好城乡环境基础设施建设,加快建设污水处理厂以及污水管网配套设施,加大农村改厕力度,推广生活垃圾“组保洁、村收集、镇转运、县处理”模式,提高城镇生活污水、农村垃圾集中处理率。

强化生态建设。深入推进绿色盐城建设,启动全国绿化模范城市创建工作,广泛开展植树造林行动。着力抓好东台、大丰、射阳3个万亩新林场建设,构筑沿海绿色屏障;抓好盐龙湖生态保护区建设,打造市区生态新亮点;抓好村庄、道路、园区、厂区、校区、社区、小区、庭院绿化建设,提高人民群众对生态环境的满意度。加强沿海、里下河湿地生态修复与保护,实施亚行贷款盐城湿地保护项目,推进自然保护区、森林公园、湿地公园等建设,打造独具特色的盐城湿地名片。加大生态市、生态县(市、区)、生态镇村创建力度,加快建设生态宜居名城。积极倡导低碳生活和绿色消费方式,形成爱护生态环境的社会风尚。

五、更加注重以人为本,努力让人民群众过上更好生活。保障改善民生是发展的根本目的。要多谋民生之利、多解民生之忧,切实增强人民群众的富足感、幸福感、安全感。

促进城乡居民增收。继续实施城乡居民收入五年倍增计划,落实更加积极的就业政策,开发更多就业岗位,加强就业援助,促进社会充分就业、高质量就业。优化创业环境,设立公共创业指导中心,建设创业培训、见习、孵化基地,实施大中专毕业生、失业人员、返乡农民工、军队退役人员创业培训行动计划,鼓励更多的人创业就业。更加关注重点人群收入增长,积极推进收入分配制度改革,健全企业职工工资集体协商、合理增长机制,着力提高中低收入者收入水平。千方百计促进农民增收,实现农民收入与城镇居民收入同步增长,并力争超过。扎实开展新一轮扶贫开发行动,对经济薄弱村、少数民族聚居村组、贫困户实行重点帮扶,帮助困难群体增收致富。

提高社会保障水平。加快社会保险扩面提标,进一步扩大住房公积金制度覆盖面,扎实开展“社会保险全覆盖街道社区”创建活动,实现城乡居民养老保险、城镇居民医疗保险、新型农村合作医疗适龄人员应保尽保。加强各类社保资金管理,确保安全增值。实施“关爱工程”,鼓励引导社会资金兴建养老机构、养老服务设施,大市区建成居家养老服务中心及信息化平台,每个县(市)建成一家300张床位以上的示范性养老机构。提高城乡低保和农村五保供养标准。加强社会救助体系建设,充分发挥红十字会、慈善总会等组织的作用,开展扶老、助残、救孤、济困、助学等活动。

繁荣发展社会事业。坚持公平均等、惠民优先,全面提升社会事业发展水平。推动文化大发展大繁荣。实施文化惠民工程,完善城乡公共文化服务设施,新建市美术馆、市民文化广场。深化文化体制改革,充分调动各类文化人才积极性,大力发展文化产业,加快聚龙湖文化产业集聚区、盐都文化产业园等13个集聚区建设。推动教育现代化创建。围绕打造重教崇文名城,进一步调整优化教育布局,实施农村中小学现代化标准学校达标工程,全市新建中小学、幼儿园32所。加强校园周边安全和校车安全,强化素质教育,提高教学水平,提升办学质量,努力让群众更满意。推动卫生服务更加便民惠民。完善基本药物制度长效补偿机制,推进公立医院改革试点,加快卫生信息化进程。抓好重大疾病防控,发展中医药事业。城南医院、市妇幼保健院新院建成使用。推动其他各项事业共同进步。进一步加强精神文明建设,倡导社会主义核心价值观,传递时代正能量。继续做好人口与计划生育工作,加快推进体育强市建设,关心支持老干部、关工委工作,积极发展妇女儿童事业,更加关爱空巢老人、留守儿童,健全残疾人社会服务体系,促进民族宗教事业规范有序发展。组织开展全国第三次经济普查工作,扎实做好科学普及、气象、防震减灾、新闻出版、档案、地方志、人事编制、机关事务管理、保密、援疆等工作。

全力维护社会稳定。深入实施社会管理创新工程,建立健全维稳工作责任落实、风险评估、矛盾隐患化解整治、督查追究等七项机制,促进社会和谐稳定。进一步加强民主法治建设,继续实施“六五”普法规划,开展法制宣传教育,推进法制文化建设,强化法律服务、法律援助,促进公正司法。加强基层社会管理和建设,大力推进社区网格化管理全覆盖,强化社会组织培育,更好地发挥基层自治组织作用。组织社会管理服务中心、信息平台、流动人口服务管理、技防城建设等重点项目攻坚,提升社会管理科学化水平。加强平安盐城建设,着力抓好社会治安、信访、环保、安全生产等工作,完善公共安全和应急管理机制,健全网络舆论引导机制、舆情监测体系,加大食品药品监管体系建设和市场整治力度,解决群众反映强烈的突出问题。加强国防动员、国防后备力量、人民防空建设,提高“双拥”共建水平,进一步巩固和发展军政军民团结。

继续办好民生实事。坚持民生优先、急需先行,进一步增加民生投入,办好20件民生实事项目。实施大市区区域供水二期完善工程,推进全市城乡区域供水全覆盖。提高市区防洪保安能力,新建、改造闸站23座,疏浚整治河道14条。优先发展公共交通,新开通公交线路5条,增加公交车辆,大市区实现公交一体化。推进保障性安居工程建设,市区新开工保障性住房7800套,竣工7500套,发放廉租住房租赁补贴2500户,让房屋被征收居民早日住上新房。加快校安工程建设,大市区加固改造、新建校舍10万平方米。加强社区卫生服务机构建设,市区新建4个社区卫生服务中心、8个社区卫生服务站。开工建设市社会福利院二期工程。整治老旧小区6个、支路巷道50条,改造建设农贸市场8个,更换新建所有老旧小区信报箱。为困难群众提供法律援助5500件以上。

六、全面提升行政效能,同心协力建设人民满意政府。今年政府工作任务重、要求高,困难多、压力大。要进一步加强政府自身建设,创造性地做好改革发展稳定各项工作。

增强政府行政能力。当前宏观形势复杂多变,对政府行政能力提出更高要求。我们要切实增强驾驭复杂局面能力,加强对经济形势的分析研判,准确把握发展走势、政策动态、市场变化,超前谋划,积极应对,努力提高组织经济工作水平。切实增强服务科学发展能力,推进服务型政府建设,深化行政审批制度改革,市级有行政审批事项的部门全部进驻政务服务中心,实行项目“一站式”办理,着力打造亲商、安商、富商的发展环境。大力强化为基层服务,深化为企业服务,细化为群众服务,推动领导干部“三解三促”、“三服务”工作常态化、长效化。切实增强解决实际问题能力,注重学习借鉴外地先进经验、成功做法,用开放的思路、创新的办法、市场的手段,破解发展难题。敢于正面矛盾、迎难而上,敢于动真碰硬、解决问题,敢于承担责任、主动作为,敢于创新实践、奋发有为,不断开创新局面,创造新业绩。

保持务实高效作风。严格执行中央和省委关于作风建设的各项规定,切实改进政风、文风、会风、作风。坚持立说立行、雷厉风行,对市委、市政府确定的重大决策部署、重点工作任务,严格按要求落实到位,确保政令畅通,提高政府执行力。整治机关“庸懒慢散”不良习气,解决推诿扯皮、敷衍拖拉等突出问题。坚持求真务实、真抓实干,大力倡导实干兴市、实干为民,真正把功夫用在注重实效、狠抓落实上,用在突出重点、突破难点上,用在推进发展、造福百姓上。坚决反对官僚主义、形式主义,坚决反对急功近利、弄虚作假,坚决反对空谈浮夸、奢侈浪费。多做打基础、利长远、惠民生的实事好事,真正使发展成果经得起实践检验,得到老百姓认可。坚持跟踪问效、督查问责,严格落实目标管理责任制和责任追究制,加强绩效管理、督查推进、效能监察、行政问责,确保各项任务全面完成。健全“四位一体”政风行风热线媒体联动机制,建立政风行风热线与“12345”热线联合督查机制,加大群众反映突出问题的解决力度。

加快建设法治政府。坚持依法行使权力、履行职责,完善行政决策程序,健全科学依法民主决策机制,使各项决策更加符合发展实际、时代要求、群众愿望。认真执行市政府工作规则和各项制度,深化扩大政务公开,完善政府信息公开、新闻发布、网络新闻发言人制度,提升政府行政权力网上公开透明运行水平,进一步规范政府行政行为。大力推进政务诚信、商务诚信、社会诚信建设。带头学法、用法、守法,提高运用法治思维和法治方式深化改革、推动发展、化解矛盾、维护稳定能力。更加自觉地接受市人大及其常委会的法律监督、工作监督和市政协的民主监督,重视新闻舆论、社会公众监督,认真办理人大代表、政协委员建议提案,不断改进政府工作。

加强勤政廉政建设。认真学习贯彻党的十八大精神,深入开展以为民务实清廉为主要内容的党的群众路线教育实践活动。坚持清廉从政、从严治政,严格落实党风廉政建设责任制、责任追究制,完善权力运行监控体系,加强领导干部任期经济责任审计,加大违法违纪案件查办力度。严格控制“三公”经费支出,降低行政成本,把更多的物力、财力用在促发展、惠民生上,提升人民群众对政府工作的满意度。

各位代表,全面建成高水平小康社会,是全市人民的共同期盼;扎实做好今年工作,是顺利实现这一目标的坚实基础。让我们按照省委、省政府和市委的部署要求,进一步解放思想、抢抓机遇,凝心聚力、攻坚克难,狠抓各项任务的落实,为实现盐城科学发展新跨越而努力奋斗!
